# The Next Twenty-Five Years in Surgery1Being the substance of an address before the Alumni Association of the Buffalo General Hospital, at a meeting commemorating the author’s completion of his twenty-fifth year in the chair of surgery.

**Published:** 1909-06

**Authors:** Roswell Park

**Affiliations:** Professor of Surgery in the Medical Department of the University of Buffalo


### BUFFALO MEDICAL JOURNAL.

Vol. Lxiv. JUNE, 1909. No. 11

### ORIGINAL COMMUNICATIONS.

### The Next Twenty-five Years in Surgery.[1]

By ROSWELL PARK, M. D., LL. D,

Professor of Surgery in the Medical Department of the University of Buffalo.

I AM not unmindful of the consideration and compliment which you have shown in thus featuring me upon this evening's program. It seems as if I never can repay my friends and colleagues who have done so much to make memorable in my life this anniversary of the twenty-fifth year of my teaching in the Buffalo Medical School.

I have been asked to give a glance into the future and to forecast what the next twenty-five years may accomplish in the domain of surgery. To illustrate the impossibility of doing this, I can only mention some of the accomplishments of the past twenty-five years that have so far exceeded our expectations as to show the futility of any such effort; nevertheless, by instituting comparisons between then and now, it may be worth while at least to make the attempt, although I shall not have the pleasure of saying at the end of the next quarter of a century,—"I told you so."

Twenty-five years ago we used to think that we had about reached the limit of what the surgeon could accomplish, yet such have been the advances made in our art and science that one may epigrammatically yet truthfully say that the impossible or unjustifiable of yesterday has become the commonplace or even the discarded practice of today. I have not infrequently made this comparison,—that among all the applied sciences none have made such rapid strides as has surgery, with the possible exception of electricity. Thus today practically no organ of the body has remained unattacked, and it would veritably seem as though, topographically at least, we had reached territorial limits. But

1. Being the substance of an address before the Alumni Association of the Buffalo General Hospital, at a meeting commemorating the author's completion of his twenty-fifth year in the chair of surgery.

here we must make a distinction between territorial limits and limitations, inasmuch as while we have brought all the organs into actual touch with the knife, we have not yet learned just how much we can safely do with them. First of all, and with the greatest probability, it may be said that what the surgeon may accomplish will depend in large measure upon the discoveries made during the same period in the physical sciences, and the extent to which he can avail himself thereof. For example, twenty-five years ago who ever dreamed of the possibilities afforded by the cathode rays or by radium. Who could have foreseen the realisation of the old philosopher's dream,—namely, the transmutation of metals; and yet, it is now known that in the presence of radium, copper changes to lithium, and then to potassium and sodium, while the emanation from radium becomes itself neon and argon (Ramsey). These may not have any bearing upon surgery, and yet, I would not like to deny the possibility of a metal suture made, say, of copper, which under the influence of radium applied externally might undergo such changes as to lead eventually to its disappearance, if this were considered worth while.

The new discoveries in physiology and biology have also the widest importance to the surgeon, and are not confined alone to conditions involving the alimentary canal.

When we know more about the actual causes of death we may be better able to avoid them. I have on another occasion coined the expression, "thanatology," which I believe to be a legitimate term that might well be made to cover a systematic study of those causes which bring about actual cessation of life, and that would be a far different study from pathological anatomy though necessarily connected with it. The surgeon then will profit by the erection of thanatology into a distinct science. When, for instance, a heart removed from the body can be made to beat more than forty hours after the death of the individual, then we have a right o raise anew the question: What really constitutes death? We have much to gain from discoveries concerning the blood and sera, the nature and causes of hemolysis, and of the various toxins and substances which produce it. This problem of hemolysis is indeed one of universal interest, but its practical bearing perhaps concerns the surgeon more than anyone else, and for quite obvious reasons, because the importance of supposedly simple transfusion of blood becomes now very great; in fact, every gain toward a more intimate knowledge of our body chemistry can but redound to the benefit of theoretical and applied surgery. Perhaps in no particular direction have we reason to hope for more than in the study of cancer, which still remains second upon the list of the ordinary causes of mortality. In this direction, if I read correctly the signs of the times, we may expect a general recognition of its parasitic character and of its infectiousness, and in proportion as these facts gain in general appreciation we shall have a more well-ordered prophylaxis, as well as much improved cannons of treatment. What we need most of all is such improvement in our powers of diagnosis as to permit an early recognition of deep-seated cancerous lesions at a time when there is still some hope for treatment, be it operative or otherwise. While I dislike to be constantly repeating my own words, this meeting furnishes another possible opportunity to make better known this most important fact, which shall serve but as an incentive to further study and as an excuse for past failure; I refer you to the fact that cancer by itself has absolutely no symptomatology of its own. Signs it has, and when the lesion is superficial these are usually unmistakable. When, however, reliable *signs* are discovered in a deep-seated lesion it is at a time when hope must be abandoned. Think it over and you will appreciate the fact not yet generally realised that cancer by itself produces no symptom, and often no collection of symptoms, by which alone it may be distinguished; *i. e.*, its symptoms are common to more than one disorder so that there is no reliable guide save the signs above mentioned, and even these too often vague. But with studies and researches such as those already published, it is not expecting too much to hope that the next quarter of a century may afford us some test of an accuracy far beyond any at present in use, by which this most infernal of all diseases may at last be mastered.

And this leads me to speak of improvements in general diagnosis which will be of common benefit to all professional mankind. No one can foresee what these may be, but you may at least rely upon it that the surgeon will be quick to avail himself of any means by which he may improve his knowledge or his skill. Thus we may profit much by a clearer realisation of that unknown relation which exists between the ductless glands and, for instance, the sexual glands, and their relations to sexuality as well as sex problems, and I beg you to notice the distinction I make between these two. In addition to these we shall realise better their relations to such nutritional defects and disorders as imbecility, gigantism, dwarfism and cretinism, rickets and osteomalacia, especially that occurring during the later months of pregnancy. The relation of all of the so-called internal secretions to these and kindred conditions are of the greatest import to the surgeon, since upon his knowledge of what may be accomplished, for instance, by opo-therapy, will depend his selection of or abstention from operative intervention.

*Operative Surgery.*—Twenty-five years ago we used to think that in this direction the limit had been about reached, and comparison of a standard textbook of 1880, with that most recently published will show, however, how much further we have gone than we then expected to go. Considering the fact that no organ of the body is now exempt from attack, it would seem as though progress in the future must be rather in the direction of improvements in technic than in extension of surgical territory.

In the *brain,* for instance, I look especially for improvement in our treatment of hydrocephalus and of lesions of the hypophysis. In the *nervous system* there will doubtless be introduced better methods of attacking the so-called neuroses and painful affections of neural origin, *i. e.,* neuralgias. In nerve transplantation and nerve grafting we have not much more to expect than improvements in technical methods, while operation for tumors and compressive lesions of the spinal cord are even now as successful as diagnostic advances permit. It is a question whether success will ever crown operations for repair of a severed cord. Should our successors succeed in this, it would certainly seem as though the very acme of surgical endeavor had been attained.

Regarding the *heart,* and remembering that it has been many times intentionally punctured by the surgeon, as well as exposed and sutured for repairs of injuries, it would seem as if there were but little left for us to improve upon. It may be that Brunton's suggestion will find favor and that some division of stenotic heart valves will be made practicable, yet concerning these I confess my own skepticism. Possibly something may be done in the matter of so-called heart-block, but not if the anti-vivisectionists have their way, since this would be a matter to be laboriously worked out in the surgical laboratory.

The surgery of the *bloodvessels* will in the future include much freer use of sutures and performance of anastomoses. Doubtless, reversals of circulation will come into play, since Carrel has already shown how perfectly they may be accomplished in animals. I have permitted my mind to perhaps wander a little and to wonder whether it would ever be practicable to connect up the carotid and the subclavian vessels, or possibly the axillary by dividing the carotid as high as possible and then carrying its end over to the subclavian in order to shunt the blood current around some intervening lesion; or whether the saphenous vein and the femoral artery might be in some way interchanged for some similar purpose.

This leads to the consideration of still more radical and, at present, more startling possibilities with regard to the *transplantation of tissues and organs* from animals to man, or even from man to man. Carrel and his co-workers have recently introduced such innovations in this direction that it is hard to predict a limit to their usefulness. Thus as showing what may be done, let me remind you of the dog into whose neck he transplanted the heart of another dog, connecting the aorta with the carotid, and the vena cava with the jugular, thus demonstrating the astonishing anomaly of an animal with two hearts beating at different rates; it would be hard to imagine anything of its kind more radical than this. But quite within the limit of human possibilities is the transplantation of a thyroid and perhaps of a kidney.

In animals, also Carrel has demonstrated the feasibility of *transplantation of an entire limb,* vessel being united to vessel, nerve to nerve, with equally careful suture of all of other tissues, and a resulting remarkable function of the new limb. He has suggested that in the human being it would possibly be practicable to apply this new method in the case of a forearm or even an arm. If so, it would far exceed, as a radical procedure, the cineplastic amputation of Vanghetti, who has recently left the tendons hanging out from the stump resulting from an arm amputation, and has later attached them to an artificial hand and forearm in such a way as to secure much more perfect control of the mechanical fingers and hand. (Illustrated on page 1048 of my "Modern Surgery.") While I have seen some of Carrel's work, and believe in it, I only know of this last procedure from the descriptions in the Italian journals. But no matter what the outcome of Carrel's work, he has already startled the surgical world with what he has shown to be practicable and of great value. It would be the greatest pity were such work as his to be estopped by any fanatic anti-vivisectionist.

We have yet very much to learn and appropriate to ourselves concerning the *ductless glands,* the hypophysis, the thymus, thyroid, tonsils and adrenals, and we might almost include the bone marrow in this list; their relations to obscure and chronic conditions, which eventually come under the surgeon's consideration, make every advance in our knowledge concerning them of importance to ourselves. Only recently have we learned to what extent their imperfect function may disable the later body welfare. The question of removal of ovaries, for instance, or of the need for transplanting them, is directly connected with these various organs, and such obscure conditions as the status lymphaticus, with all its possibilities of danger, with or without operation, are inseparably connected therewith. Various internal conditions hinder us largely in some of the work which we might otherwise carry out—namely, their inaccessibility, and the internal arrangement of their bloodvessels which make even satisfactory readjustment next to impossible.

Regarding the improved surgery of the *lungs* there is yet a field for considerable advance. Since certain Italian investigators have virtually abandoned their practice of exsection of portions of the lungs, this procedure has fallen into almost complete shadow, and yet there is more present reason than ever for experiments in this direction. Now that opening the thorax has been shown not to be such a formidable procedure as it was formally considered, there seems to be ample justification for removal of a portion of a lung, either for malignant disease, for tuberculosis, or some other and extremely rare condition, providing only that the morbid process is limited within reasonably strict boundaries. In Italy, this measure dropped into disfavor some twenty-five years ago after one of the Italian surgeons who had demonstrated, as did I for myself about the same time, the large percentage of success which followed removal of a portion of the lung in dogs. Completely satisfied by these results he removed the apex of the left lung of his fiancée because of localised tubercular disease. Her death so upset him that he committed suicide, and it seems to have discouraged others as well, so that we seem to have heard of nothing of this character in very late years. Notwithstanding his sad experience, there is a field open in this direction, in which I am inclined to think the more adventuresome surgeons will work again.

More promising and less hazardous, although in appearance more formidable, is the method of Friedreich, of Marburg, who has lately apparently been curing certain cases of tubercular disease confined to one lung by producing an artificial stasis by compression, removing for the purpose practically all the ribs on one side, and permitting compression of the enclosed lung by atmospheric pressure. For this purpose he raises a "U" shaped flap from the entire side of the thorax and then removes the ribs subperiosteally without opening the pleural cavity. And he has had a degree of success which stamps the procedure as being justifiable and hopeful providing only that the disease is limited.

All of which raises the question concerning the utility of the pneumatic cabinet in these extensive operations where the thorax is opened. In thoracotomy it is theoretically advantageous; in actual practice, however, it is scarcely indicated, and will probably never come into general use, not at least in its present form; moreover, it has been shown that it is not a necessity, and that if any apparatus be required it can be found in some simpler form such as was employed in Buffalo, first by Professor Dalton, and later by Dr. Fell. It is quite possible to make this in such form and to so use it as to accomplish all that is done with the cabinet, and in a vastly simplified manner.

Turning our attention next to the *abdomen,* and speaking first of the stomach, we may say that what may be accomplished in the future with this organ will depend in large measure on what improvements are made in diagnosis. It would also seem as if the technic of stomach operations were now practically complete, since formally cumbersome methods have been simplified, and novelties can only be introduced when dealing with some most rare or peculiar condition. While it has been shown to be possible to reach the esophagus through the posterior mediastinum, by a rib resection near the spine, and while it has been proposed to make a practically artificial esophagus, or to externalise it, the cases which demand such measures, or the men who are competent to perform them, will be each exceedingly rare. Therefore what we most need now is not so much technical improvements as an opportunity to apply our present technic at a much earlier period than has hitherto been possible. Did time permit I would like to digress and insert a plea for early exploration in cases of suspected gastric ulcer and cancer; especially in the latter is early diagnosis particularly at fault or impossible, and by the time it has become easy the period of the surgeon's possible usefulness has too frequently passed; hence my plea that a suspicion of gastric carcinoma, or for that matter of any internal cancer, not only justifies but should make imperative an early operation, first for diagnostic purposes, and secondly, in order that radical measures may be carried out at a time when there is about them something of real promise.

All that has been said of the stomach is virtually true of the *intestines,* and to speak further of them here would be simply to reiterate.

With regard to the *urinary organs* there is still much to accomplish, including always improvements in diagnosis. The ideal operation for movable kidney is perhaps yet to be made known to us, and the question of nephrotomy or nephrectomy in tubercular lesions is still discussed by many, although for my own part I do not hesitate to advise the latter as the better of the two. Capsulotomy for Bright's disease is still under discussion. It has suffered from this feature that the physician will scarcely ever turn his patient over to the surgeon for this purpose until it is far too late. Here, as in many another way, surgery has suffered in reputation because the right thing has been done at the wrong time, *i. e.,* too late; this has led the laity into prejudice against operative intervention, a prejudice quite undeserved, and made to redound to our discredit. For my own part I believe in incision and removal of the capsule, and to such an extent that I practically do it in every operation upon the kidney. It offers much by mere relief of tension, and consequently of pain and improvement of function, while there can be no surgical objection to it. As to the matter of what it may accomplish beyond this, I do not care to enter into discussion at this time. There is apparent opportunity for transplantation, and it would not be at all surprising to see this measure placed upon a sound footing within the next twenty-five years.

With the *ureters* much is yet to be accomplished, partly in the nature of their diversion for various purposes, and partly in the nature of operation for their intrinsic diseases. Thus they have been frequently diverted into the rectum for ectopia of bladder, into the vagina, or out upon the back, as suggested by Watson, while I have myself grafted them into the appendix. Regarding all, or nearly all of these operations, we need and shall gain clearer indications and improved methods.

Concerning the *bladder* we may look especially for those improved methods which we sadly need in treating extroversion or ectopia, concerning which it may be said that there is scarcely any condition susceptible to surgical treatment which needs it more and promises less than this particular bête noir of the surgeon. It seems to me that externalisation will be more frequently performed than has hitherto been the case. And I look forward, also, to improved methods and more frequent performance of partial or even complete exsection of the urinary bladder.

The *prostate* is at present the field of great activity among the surgeons. What is especially needed now is such a popularisation of its exsection as to lead to its more popular and earlier resort. I look, also, for a final recognition of the superior advantages of the suprapubic method of removing it, save in those instances where the indications are clearly in favor of the lower route. I hardly need say much here about the *female pelvic organs,* save, perhaps, to express a hope that the future will show a diminished number of operations because of disturbances therein, but we do very much need a clearer recognition regarding, especially, the functions of the ovary, and in this respect I might include also the testicles, because their influence upon nutrition is as positive as it is yet ill understood. Transplantation of the testicles has not yet come into vogue, but transplantation of the ovaries has been shown to be practicable and even remarkably successful. Certainly in the next quarter of a century, experimenters should settle the question whether it be practicable to transplant into any convenient portion of the body the testicles either of animals or man, and not only to thus remove them but to preserve their function. This question once experimentally and favorably settled, practical application will find an ample sphere of usefulness.

My time is short and I will but allude to possible improvements in *orthopedics,* and the surgery of *bones and joints.* We need more positive indications for operations upon the spine, whether the cutting methods or the bloodless operations of Calot be considered. These may be regarded as scarcely emerged from the experimental stage, but operations for various paralytic deformities are now upon such a firm basis that what is needed is mainly the spread of knowledge concerning their value. These operations at present are mainly practised by a limited number of expert orthopedists, and by a few general surgeons. Cases demanding them are, however, so numerous, that they need to be popularised, and this will surely happen within the next twenty-five years. During this period also may be yet looked for improvements in the surgery of the tendons, and particularly of the muscles; preceding or along with these will be also a better realisation of processes of repair and complete restoration of function in these tissues. Moreover, along with all the tendon surgery will come an appreciation of the benefits to be gained in certain cases by nerve transplantation and nerve grafting.

We may expect, also, certain improvements in our operations upon the *joints,* especially in the nature of securing motion after excisions. And we may anxiously look for, and with reasonable confidence expect, enlargement of our knowledge of the infecttious arthritides, and in repairing or atoning for their disturbing consequences.

Once more I must beg you to realise the difficulties before the man who aspires to play the rôle of prophet, and to be lenient in your judgment on such prophecies as I have permitted myself tonight to make. But there is one final consideration, which concerns the welfare of the entire profession, upon which I do not hesitate to speak with positive convictions, and to the following effect. The whole aspect of the practice of surgery twenty-five years from now will depend upon our management of the two greatest dangers which at present menace it, and without going into detail or saying anything more concerning this, upon which I feel very deeply, I would simply lay them before you. The first of these dangers comes from incompetence and *too much eagerness* on the part of those who essay to do surgical work, and the second comes from a *lowering of professional standards;* the *introduction into our science and art of debasing principles of commercialism.* And in this I name especially the system of spoils or graft, or whatever you like to call it, which is usually spoken of in lighter terms as division of fees. Let us hope that standards in both these directions will be raised rather than lowered, and let us hope that higher aspirations and determination for improvement will dominate and triumph in the end.

430 DELAWARE AVENUE.

